# COVID-19 induced restriction in developing countries and its impacts on pollution load: case study of Lagos mega city

**DOI:** 10.1016/j.heliyon.2022.e10402

**Published:** 2022-08-27

**Authors:** E.L. Odekanle, B.S. Fakinle, O.J. Odejobi, O.E. Akangbe, J.A. Sonibare, F.A. Akeredolu, O.M. Oladoja

**Affiliations:** a Department of Chemical and Mineral Resources Engineering, First Technical University, Ibadan, Oyo State, Nigeria; b Department of Chemical Engineering, Landmark University, Omu-Aran, Kwara State, Nigeria; c Department of Chemical Engineering, Obafemi Awolowo University, Ile-Ife, Nigeria; d Department of Agricultural Engineering, First Technical University, Ibadan, Oyo State, Nigeria; e Department of Computational and Mathematics, First Technical University, Ibadan, Oyo State, Nigeria

**Keywords:** Air pollution, COVID-19, Air quality, Anthropogenic activities, Lockdown

## Abstract

Sudden outbreak of COVID-19 pandemic globally in 2020 warranted urgent course of actions to guide against its escalation. The first and immediate measure adopted by several nations was the imposition of restriction on transport, industrial, commercial and social activities; and this step has thus, provided a platform for the impact assessment of the restrictions on ambient air quality, especially in developing nations such as Nigeria. The levels of four criteria air pollutants (PM_2.5_, SO_2_, NO_2_, and PM_10_) in ambient air of Lagos city before, during and after the restriction periods were compared to establish the extent of change caused by the restrictions. The results revealed a decline of 74.0, 79.7, 55.0 and 58.5% in the levels of SO_2_, NO_2_, PM_2.5_, and PM_10_, respectively during the lockdown period. The results also revealed that, despite the huge reduction in the atmospheric emissions witnessed during lockdown period, air quality within the region was still poor, as the levels of most of the pollutants were above the recommended limits. These findings suggested that apart from the restricted activities, there are other air pollution sources within the city which increased the pollution load in the ambient air. Conclusively, while the restriction led to untold economic hardship, it equally enhanced quality of ambient air. Cleaner technology is advocated to ensure reduction in the consumption of fossil fuel instead of the common practice of end-of-pipe technology, for environmental sustainability.

## 1 Introduction

**Figure 1:**
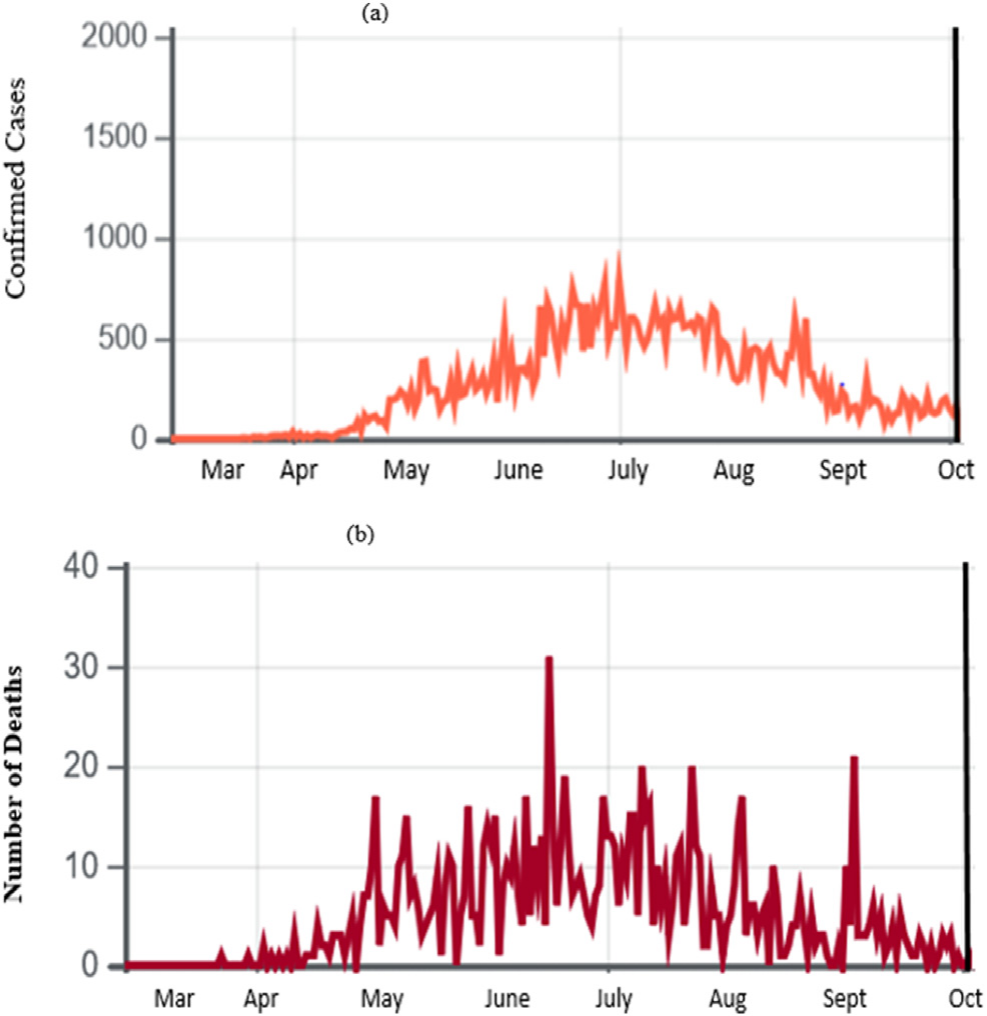
Curve showing (a) daily confirmed and (b) deaths cases of COVID-19 from February to October, 2020 (NCDC, 2020).

First confirmed COVID-19 case was reported in the city of Wuhan China, on December 29, 2019 (Huang et al., 2020; Zhou et al., 2020a, Zhou et al., 2020b) after which the virus spread across to other nations of the world. It was reported that close to twelve million confirmed cases of the disease, including over five hundred thousand deaths, were since recorded, with United States of America having more than 2 million cases and well over one hundred thousand deaths (WHO, 2020). Countries like Brazil, Russia, UK, Italy, France, Spain and Mexico also had their own large shares with each of these countries having several hundreds of thousands of confirmed cases and varying proportion of deaths (Sabyasachi and Nitin, 2020). COVID-19 has been adjudged as the greatest challenge faced by human race after the Second World War (Gautam et al., 2022). At the advent of COVID-19, developing nations especially African countries appeared to be most vulnerable to the pandemic, considering the continents populations and the resulting challenges against social distancing. The first COVID-19 case in Africa, was reported in February 14, 2020 in Egypt; and by May, same year, the pandemic had spread to fifty-four African countries including Nigeria. Nigeria had her first case reported on February 28, 2020 in Lagos city, after the earlier reported case in Algeria in February 17, 2020 (WHO, 2020). Most countries in the continent swiftly reacted with the enforcement of border closure, quarantines and lockdown. Nigerian Government, having inaugurated committee on COVID-19, announced immediate lockdown in three major cities in Nigeria (Lagos, Abuja and Port Harcourt) on March 29, 2020 with imposition of restrictions on all transport, industrial, commercial and social activities. Schools, recreation and religious centres were shut down with enforcement of the use of face mask and maintenance of social distancing. The lockdown which took effect at 11: 59 p.m. on March 30, 2020 was for initial period of fourteen days. However, continuous increasing cases extended the lockdown till September 25, when the lockdown was totally relaxed due to some observed drops in the level of cases earlier in the month as reported by the Nigeria Centre for Disease and Control (NCDC). Figure 1 shows the daily COVID-19 confirmed cases and deaths from March to October, 2020.

During this period of six months, the number of confirmed cases escalated to a total of fifty-eight thousand and sixty-two (58,062). Lagos recorded 19,174 confirmed cases which was about 33% of the total recorded cases in the entire country. Regardless of impacts of COVID-19 on Lagos, it is still the economic focal-point of Nigeria, and one of the megacities with the fastest growth in the West Africa (Hoornweg and Pope, 2016).

Air pollution continues to attract scientific attention in recent years due to its impacts on both environmental and human health. It has been reported that 91% of world population reside in environments where air quality fall below World Health Organization's recommended limits (Abulude et al., 2021, 2022). Globally in 2016, 4.2 million premature deaths were reported to have been caused by air pollution-related illness; out of which 91% were from developing countries (WHO, 2018; Abulude et al., 2022). Environmental quality in Nigeria is becoming worrisome, possibly because of the growing rate of population and lack of sound environmental management policy. Report has it that Nigeria is ranked 152nd on the World Risk Index for air quality (Abulude et al., 2022) and there is possibility of continuous decrease in Nigerian's ambient air quality as population growth rate and anthropogenic activities increase; Akinwumiju et al., 2021).

Serious ambient air pollution problem in Lagos could be traced to high levels of nitrogen oxide (NO_X_), sulfur dioxide (SO_2_), particulate matters (PM_2.5_ and PM_10_), Ozone (O_3_) and air toxics which can pass through lung barriers and get into blood streams thereby causing respiratory and cardiovascular diseases resulting into premature death (World Bank, 2019).

The major sources of these pollutants are anthropogenic in nature, and these include motor vehicles, industrial emission and residential power generation (generators) among others. Report has it that high pollution level in Lagos mega city has been attributed to, among others, large volume of aged diesel and gasoline vehicles ((227 vehicles/Km/day) with high sulfur content (Odekanle et al., 2017). This pollution concentration in Lagos has also been reported to reach 68 μg/m^3^, similar to the pollution level in other megacities like Beijing and Cairo (World Bank, 2019); and this accounted for more than eleven thousand premature deaths (the highest in West Africa) for which children of under five years of age are most affected accounting for 60% of the death (World Bank, 2019).

In furtherance to research attempts to examine atmospheric pollution load from anthropogenic activities, this study explores the effects of movement restrictions due to COVID-19 pandemic on quality of ambient air in African nations, using Lagos city as a case study. Some studies on the effect of total lockdown due to COVID-19 pandemic on air quality across the globe have been reported, indicating various degrees of decline in the air pollution levels in their respective study areas. In India, effects of controlled emission on the quality of atmospheric air during the pandemic era was investigated and improved air quality was reported with reduced pollution levels ranging from 10% to 53.61% (Sharma et al., 2020; Kabiraj and Gavli, 2020; Singh, 2021a). Study by Ambade et al. (2021) showed that COVID-19 lockdowns significantly reduced the concentrations of Black carbon, PAHs and PM_2.5_. Other similar studies also reported a declining trend in the atmospheric concentrations of PM_10_, PM_2.5_, SO_2_, NO_2_, and CO in eight most polluted Indian cities during the COVID-19 pandemic (Gautam et al., 2021a, b); while strong relationship between the concentrations of the atmospheric pollutants, meteorological factor and COVID-19 cases has also been established (Gautam et al., 2021c). Also, Selvam et al. (2020) examined possible air quality improvement arising from the shutdown of industrial operation and restriction on vehicular movement; a drop of 30–84% pollution levels was reported. One month into the lockdown, air quality in Barcelona (Spain) was reported to improve by 51 % NO_2_ and 31% PM_10_ (Tobias et al., 2020). Decrease in the primary emission of pollution in China during lockdown was reported by Huang et al. (2020), while in Delhi (a mega city like Lagos), at least 31% reduction in National Air Quality Index was observed across the city (Mahato et al., 2020). Periodical lockdown as a tool to mitigate air pollution was suggested by Srivastava et al. (2020) when the study observed a sharp reduction in air pollution indices in Lucknow and New Delhi, India within 21 days of lockdown, while it has also been shown that despite the decline and hardship on global socio-economic growth, the merit of COVID-19 lockdown lies in its potential to restore some of the lost natural conditions of our environment (Thapliyal et al., 2022).

Literature review showed variation of air pollutants over several developing countries during COVID-19 era. It is thus obvious that there is scantiness of reported information on the restriction effects of the pandemic lockdown on the quality of environmental air in Africa continents. The reported information on other continents may not give the true reflection of the quality of air in African sub-region. It is therefore pertinent to explore the pandemic lockdown effects on the health of environmental air within the continent of Africa. Lagos is considered as a case study considering its population in sub-Saharan Africa (and the accompanying challenges of social distancing measure) as well as its industrial and economic importance to African continent. The uniqueness of the study lies in on-site measurement of the pollutants during the lockdown season which was then compared with the historical levels of air pollution before and after COVID-19 pandemic eras (with equal spread of monitoring period). The study will complement the existing studies on air pollution by providing helpful information about the conditions of the atmospheric air and the effects of COVID-19 lockdown on air quality. This will enhance understanding of the impacts of human activities on environmental air. It will also provide information for policy makers on the understanding of the changes in the level of air pollution, which can give insight into how to achieve improvement in air quality, especially when there are enforced restrictions on the emission from various sources.

## 2 Experimental

### 2.1 Study area

Lagos, the capital city of Nigeria from 1914 to 1999 lies between latitude 6.22^o^–6.42^o^ N and longitude 2.42^o^–3.22^o^ E in the South-western Nigeria and bounded in the north and east by Ogun State, west by Republic of Benin and the south by Atlantic Ocean. Its vegetation is partly rainforest and swamp mangrove with dry (November–March) and wet (April–October) seasons. It has bi-modal rainfall pattern with average rainfall of 2000 mm and elevation of less than 15 m above sea level (Fuwape et al., 2020). It is situated in coastal area with average wind speed and relative humidity of 4.3 km/h and 79%, respectively; as well as monthly mean temperature range of 23–32 °C (Komolafe et al., 2014; Njoku et al., 2016). Lagos has some peculiarities it presents: The city is characterized with the presence of more than 300 industries (Njoku et al., 2016) which contribute in no small measure to the increased atmospheric pollution load of the area. Also, it is the most populated urban centre in Nigeria with most serious air pollution problems (Odekanle et al., 2017) emanating from large volume of vehicles and high traffic density. The choice of Lagos city as a case study is based on some criteria. It is the most populated city in Africa with an estimated population of 21 million and annual growth rate of 2–3%; followed by Cairo in Egypt and Kinshasa in Congo (Worldatlas, 2019). The city remits well over 55% of the country's value-added tax and also serves as economic hub to other African countries. Also, African nations, such as Nigeria will possibly overtake United Nation on population chart by the end of the century, Lagos population is therefore considered a major factor if this would be realized.

### 2.2 Material and sampling

In this study, passive air samplers were developed and employed for NO_2_ and SO_2_ sampling. Several studies have employed and described the use of passive samplers for monitoring of gaseous pollutants (Gül et al., 2011, Demirel et al., 2014, Can et al., 2015, Artun et al., 2017). The developed sampler consists of a plastic cylindrical tube (of length and inner diameter of 2.5 cm and 2.0 cm, respectively), mesh barrier made of stainless steel, plastic close cap, fixed plastic ring (5 mm) and filter paper impregnated with specific solution. Glass fibre filter paper (Whatman GF/A) already soaked in 20% aqueous solution of Triethanolamine was used to collect NO_2_ and SO_2_. The dried filter papers were located at the at the base of the sampler and stationed with the 5 mm plastic ring, while the inlet ends were sealed with a plastic closed cap.

During the sampling period, the impact of the turbulence of the wind inside each sampler was reduced by attaching the mesh barrier to an open end of the sampler (though the barrier was replaced with closed cap while commuting the sampler to the sampling locations). Also, at the sampling locations, the passive samplers were placed in a shelter and hung on a pole in order to prevent the possible negative effects of meteorological factors (wind velocity, rain etc). Before the analysis, used filter papers were withdrawn from the sampler and their extraction was done using a mixture of 10 ml of deionized water and 0.004 ml, 30% solution of hydrogen peroxide (H_2_O_2_) for about 900 s. Analysis of these samples were carried out using DIONESS ICS-3000 ion chromatography. Determination of concentrations of the measured pollutants followed Fick's law of diffusion (Gorecki and Namiesnik, 2002).

Similarly, passive samplers were developed for the samplings of particulate matters following the method described by Gaga et al. (2012). A 39 mm cyclone aluminium and impactors were connected to pump (SKC, Inc, USA) which sucked air at a flow rate of 2.5 L min^−1^ over a Teflon Filters for the measurement mass concentration of PM_2.5_. Mass concentrations of PM_10_ was determined using a stage impactor ((SKC, Inc, USA) at a flow rate of 3.5 L min^−1^. The weights of the Teflon filters were determined prior to and after the sampling period using Kerro (BL5002) digital weighing balance, while the flows were determined prior to and after each sampling period using calibrated rotameter.

Pre-lockdown (PRLD) sampling commenced in December, 2019 and lasted till February, 2020; lockdown (LD) sampling period was from May, 2020 to July, 2020 while post lockdown (POLD) sampling commenced in October, and spanned through December, 2020. To prevent meteorological influence on sample collection, all sampling location points were visited at every twenty –four hour for three months during each sampling period. The selected sampling locations were typical of the residential, commercial and industrial vicinities of Lagos. Statistical analysis was done with the aid of Statistical Package for Social Science (SPSS, 17.0).

### 2.3 Effects of meteorological variables on pollutants’ levels

Relationship between the pollutants and the surrounding climate was also established by estimating corelation matrixes between the pollutants and metrological variables (wind-w_n_, relative humidity-Rh and mean temperature-T_av_). Cross correlation analysis was used to check significant relationship between the time series of the pollutants and the metrological variables, and regressed using step-wise regression procedure with MINITAB 17.0 version to develop models represented by Eq. (1) for the three periods

1 $$\gamma_{c}=\;\beta_{o}+\overset{n}{\underset{i=1}{\sum }}\left(\beta_{n}X_{n}\right)+e$$

where Y_c_ is the pollutant, X_n_ (n = 1, 2....) are the significantly correlated parameters i.e. (climatic variables), βo is model constant, β_n_ is the coefficient of respective variables and e_i_ is the error term. The models developed were validated for reliable prediction using split sample method where available datasets were randomly split into halves. First dataset sample was used for calibration while the other set was used for validation. The statistical metrices used to evaluate the developed models Mean Absolute Error, Coefficient of Correlation and Mean Bias Error (Sayegh et al., 2014; Akangbe et al., 2018).

### 2.4 Air quality index

Air quality index (AQI) was also used to assess environmental air quality during the monitoring periods. The sub-index for each pollutant (I_p_) was first calculated using Eq. (2) (United State Environmental Protection Agency, US EPA, 2018)

2 $${\text{I}}_{\text{p}}=\frac{I_{H0}-I_{Lo}}{BP_{Hi}-BP_{Lo}}\;\left(C_{p}-BP_{Lo}\right)+I_{Lo}$$

where I_p_ = the sub-index for pollutant p, Cp = the truncated concentration of pollutant p, BP_Hi_ = the concentration breakpoint that is greater than or equal to Cp, BP_Lo_ = the concentration breakpoint that is less than or equal to Cp, I_Hi_ = the AQI value corresponding to BP_Hi_, I_Lo_ = the AQI value corresponding to BP_Lo_.

A high AQI is an indication that the level of the air pollutant will not only have adverse effect on humans’ outdoor activities but also pose high threat to human health. To evaluate the AQI for SO_2_ and NO_2_, the concentrations of these pollutants were extrapolated for 1-hour averaging periods using atmospheric stability formula (Bashar et al., 2009; Fakinle et al., 2013) given as by Eq. (3):

3 $$C_{1}=C_{0}{\left(\frac{t_{0}}{t_{1}}\right)}^{n}$$

where $C_{0}$ = measured concentration; $C_{1}$ = extrapolated concentration; t_o_ = observed time; t_1_ = standard time (depending on the averaging time of the guideline); n = 0.28, stability dependent exponent.

### 2.5 Quality control/quality assurance

Field blanks were frequently taken and analysed to assess any contamination of the sampler while commuting the samplers to the sampling locations. Subtraction of the blank levels from the measured results was done if the concentration of the blank was higher than 10% of the analyte (Gaga et al., 2012). Each sampling location had duplicate measurements and duplicate measurements with more than 25% difference were ignored. The quality of the sampler for NO_2_ and SO_2_ was earlier assessed by comparing its result with Multiplus automated gas analyser and the percentage relative error was less than 12%. During the analysis, NO_2_ and SO_2_ were analysed as ${\text{NO}}_{2}^{-}$ and ${\text{SO}}_{4}^{2-}$ respectively. The limit of detection (LOD) was evaluated by analysing the second lowest calibration standard repeatedly for ten times and the standard deviation of the measurement was multiplied by three. Limit of detection of ${\text{NO}}_{2}^{-}$ and ${\text{SO}}_{4}^{2-}$ are 0.003 and 0.25 ug/l respectively. Also, analysis precisions were estimated as coefficient of variance using five replicate measurements of the intermediate standard solution (Üzmez, 2018). Obtained coefficient of variance values for ${\text{NO}}_{2}^{-}$ and ${\text{SO}}_{4}^{2-}$ were 4.1 and 3.9% respectively. Concentrations of PM_2.5_ and PM_10_ were obtained as the difference between the pre-weight and post weight of the Teflon Filters. Teflon filters were refrigerated for at least 24-hour before and after sampling to be measured on the weighing balance. Each filter was weighed three times at interval with the assurance that the differences in the values must not be greater than 0.005 mg otherwise, the values were rejected and the sampling repeated. The gain in weight was estimated and the obtained data was corrected for the field blank weights. Furthermore, PM_2.5_ and PM_10_ concentrations obtained from the passive sampler were compared with readings from Met One Aerocet Mass Particle Counter and the percentage relative error was obtained to be less than 15% (Özden, 2005).

## 3 Result and discussion

### 3.1 Statistical analysis

The statistical analysis of the levels of the pollutants before, during and after the restriction periods are presented in Table 1:

**Table 1:** Statistical analysis of the concentration of the pollutants.

Pre-Lock Down Period					Lock Down Period				Post Lock down Period			
	SO_2_	NO_2_	PM_2.5_	PM_10_	SO_2_	NO_2_	PM_2.5_	PM_10_	SO_2_	NO_2_	PM_2.5_	PM_10_
Min.	0.40	0.10	84.5	54.5	0.00	0.00	42.4	94.6	0.40	0.06	75.9	201.3
1st Qu.	0.60	0.15	163.6	319.5	0.10	0.01	53.3	123.5	0.60	0.10	133.2	321.8
Median	0.80	0.20	192.3	371.1	0.20	0.05	53.3	123.5	0.80	0.15	151.6	412.9
Mean	0.76	0.23	187.3	376.1	0.19	0.05	66.2	162.9	0.74	0.18	165.4	378.6
3rd Qu.	0.85	0.30	221.9	466.3	0.30	0.08	76.6	192.8	0.80	0.23	195.6	431.2
Max.	1.20	0.40	261.1	641.2	0.40	0.20	91.3	305.9	1.00	0.40	281.4	532.4
St. Dev.	0.19	0.01	47.3	114.7	0.15	0.04	15.0	50.2	0.17	0.09	48.6	85.5
Percentage	**76.48**	**50.12**	**44.7**	**40.9**	**5.00**	**10.51**	**15.8**	**17.7**	**18.52**	**39.36**	**39.5**	**41.3**

Note: SO_2_ and NO_2_ are in ppm while PM_2.5_ and PM_10_ are measured in μg/m^3^.

During lockdown period, reduced concentrations of the pollutants were observed. There were times SO_2_ and NO_2_ were undetectable and this observation could be attributed to the absence of both vehicular movements and industrial operations, which are major air pollution sources within the city. The highest levels of these pollutants were observed before the lockdown when all the anthropogenic activities were going on undisturbed. Exhaust emissions from vehicles using fossil fuel are major sources of nitrogen oxide (in form of NO_2_) and sulphur oxide (in form of SO_2_) (Pratama et al., 2019) and Lagos city, with its characteristic high vehicular volume experienced high level of the pollutants during this period. For PM_2.5_, its minimum concentration (42.4 μg/m^3^) was recorded during lockdown period, while maximum concentration (281.4 μg/m^3^) was recorded after the lockdown. Although, reduced concentrations of PM_10_ were recorded during lockdown, its lowest and highest concentrations were recorded before the lockdown. The concentrations of particulate matters (PM_2.5_ and PM_10_) recorded in this study are favourably comparable to the concentrations recorded for the same pollutants within the eight most polluted cities in India (Gautam et al., 2021a). SO_2_ and NO_2_ levels in this study are lower that several of the previously documented studies (Srivastava et al.., 2020; Gautam et al., 2021a; Lian et al., 2020). The difference is thought to be due to the differences in level of industrialization and vehicular volume between the study locations as these pollutants are more pronounced in industrial centres. 76.48 % of total SO_2_ concentration was recorded before the lockdown, while this percentage was drastically reduced to 5.00 and 18.52 % during and after lockdown periods, respectively. Similarly, of the total NO_2_ concentration recorded for the entire three sampling periods, 50.12 % was recorded before lockdown; 10.12 % during lockdown and 39.36 % after lockdown. Similar trends were noticed for PM_2.5_ and PM_10_. Previous studies reported comparable reductions in the percentage of these criteria pollutants during the period of restriction (Sharma et al., 2020; Selvam et al., 2020). It could therefore be said that COVID-19 induced lockdown was responsible for the lower level of the pollutants compared to what was recorded before and after the lockdown periods when no restrictions were placed on all human activities and thus, an indication that human activities had strong influence on environmental air quality.

In this study, mean concentrations of PM_10_ of 367.1, 162.9 and 378.6 μg/m^3^ recorded for the three respective sampling periods were above the USEPA regulatory limit of 150 μg/m^3^. Apart from this, concentrations for both pre-lockdown and post lockdown (367.1 and 378.6 μg/m^3^) were higher than the Nigerian Federal Ministry of Environment (FMEnv) permissible limits of 250 μg/m^3^ (Federal Environmental Protection Agency, FEPA, 1991). PM_2.5_ Mean concentrations of 187.3, 66.2 and 165.4 μg/m^3^ were equally higher than USEPA and WHO recommended limits of 35 (USEPA, 1995) and 25 μg/m^3^ (WHO, 2006), respectively but fell below FMEnv limits (FEPA, 1991). High value of PM_2.5_ may be connected to the exhaust emission from vehicles due to high traffic and emission from fuel combustion from diesel or petrol generator. In Nigeria, the frequent power outages from the national grid have made the use of generator a common practice both in residential and industrial areas. The average concentrations of SO_2_ recorded were 0.76, 0.19 and 0.74 ppm for pre-lockdown, lockdown and post lockdown periods, respectively. These concentrations were above the United State Environmental Protection Agency (US EPA) and National Emission Standards for Hazardous Pollutants (NAESHAP), regulatory thresholds of 0.1 ppm (US EPA, 1995; NAESHAP, 2021). SO_2_ inhalation has been associated to an aggravated risk of lung cancer mortality (Cao et al., 2011), increased respiratory challenges and premature death (Obanya et al., 2018; Orellano et al., 2021). SO_2_ released in the atmosphere is also a precursor for acid rain and particulate build-up in the atmosphere (Subodh 2017). Also, the mean values of NO_2_ recorded during pre-lockdown period exceeded 0.2 recommended by World Health Organization, though values obtained during other sampling periods were below permissible limits (WHO, 2006; FEPA, 1991; USEPA, 1995). Short-term exposure (ranging from 30 min to 24 h) to NO_2_ has been reported to induce respiratory issues, especially in asthmatic patients (Mandel et al., 2015). Generally, reduction of air pollutants is critical to both environmental and human health. For instance, inhalation of particulate matter has adverse effects on human health as this kind of pollutants has potential to easily reach the deep part of the lung, thereby causing respiratory diseases (Mandel et al., 2015). PM_10_ and PM_2.5_ are known as thoracic and fine particles, respectively. Exposure to them are hazardous due to the possibility of adsorption of harmful contaminants (e.g lead and mercury) on the surface of these pollutants (Obanya et al., 2018). PM_2.5_ also has the potential of increasing the risk of cardiovascular diseases (Uboh et al., 2017).

The percentage changes in the pollutants ’concentrations during the periods under consideration are presented in Table 2. There was a high percentage reduction of all the pollutants during lockdown with NO_2_ having a percentage reduction of almost 80%. This reduction is higher than what was reported by Fuwape et al. (2020) and Tobias et al. (2020) but comparable to the report of the study conducted by Selvan et al. (2020). Similarly, there was a gradual increase in the emissions of air pollutants after the lockdown, when compared with what was recorded prior to COVID-19 lockdown (except PM_2.5_) which showed percentage reduction of 2.83%. This observation could be attributed to the absence of industrial, commercial and vehicular activities within the city during the lockdown period.

**Table 2:** Percentage Changes in the air pollutants’ concentrations.

	Pre-lockdown-lockdown	Pre-lockdown-Post lockdown	Lockdown-Post lockdown
SO_2_	−74.0	+1.03	289.0
NO_2_	−79.7	+1.5.9	362.0
PM_2.5_	−55.0	−2.8	116.0
PM_10_	−58.5	+5.89	115.0

Lagos, being a densely populated environment, anthropogenic activities, which are thought to be the major sources of air pollution within the city were being carried out without interference prior to lockdown. However, as the lockdown commenced and progressed, all these activities were suspended and there was a continuous decrease in the emission of the pollutants; the result of which was seen in the reduced pollutants’ concentrations in the ambient air quality. On the other hand, the first three months of lifting the ban on all movements in Lagos city (first three months of post lockdown), witnessed an exponential percentage increase in the concentrations of the air pollutants with gaseous pollutants, NO_2_ and SO_2_ having 362 and 289 % increase respectively. The astronomical percentage increase could be attributed to the full resumption of activities immediately after the lockdown. Considering the effects of the lockdown on the economy of the indigenes, there was eagerness from all sectors of economy to make up for the lockdown periods, hence commencement of full industrial and commercial activities, as well as heavy vehicular and pedestrians traffics. Fuwape et al. (2020) has reported similar observation in the air quality in Port Harcourt and Abuja cities during lockdown.

### 3.2 Effects of metrological variables on pollutants’ levels

To have more comprehensive understanding of the effects of lockdown on environmental air, satellite data (Table 3) obtained from World Weather Forecast was compared with ground data. From the satellite data, it could be observed that nitrogen was not detected until after the lockdown was lifted. Apart from this, the concentrations of the pollutants as captured by the satellite were relatively lower than those measured at ground stations (Table 1). This could be attributed to distance of the satellite to the emission sources and thus, diffusion of gases (which involves movement of gases from regions of elevated concentration to region of lower concentration) was obeyed.

**Table 3:** Statistical analysis of the concentrations of the pollutants obtained from the satellite.

Pre-Lock Down Period					Lock Down Period				Post Lock down Period			
	SO_2_	NO_2_	PM_2.5_	PM_10_	SO_2_	NO_2_	PM_2.5_	PM_10_	SO_2_	NO_2_	PM_2.5_	PM_10_
Min.	0.21	0.00	0.0	25.0	0.57	0.00	8.0	13.0	0.85	0.00	8.0	13.0
1st Qu.	1.40	0.00	29.5	51.9	1.07	0.00	10.3	17.6	1.82	2.04	28.8	47.1
Median	1.91	0.00	35.1	61.4	1.32	0.00	10.9	19.4	2.02	3.29	32.2	56.6
Mean	2.03	0.00	36.8	64.3	1.43	0.00	11.1	19.6	2.03	2.99	32.3	54.9
3rd Qu.	2.36	0.00	4.8	73.9	1.67	0.00	11.7	21.3	2.23	4.04	36.4	62.1
Max.	8.60	0.00	108.0	187.0	4.43	0.00	10.3	17.6	3.26	5.57	60.0	105.0
St. Dev.	1.08	0.00	13.9	23.1	0.59	0.00	1.3	3.3	0.43	1.50	8.4	16.8
Percentage	**57.25**	**0.00**	**60.6**	**60.4**	**9.40**	**0.00**	**5.7**	**5.7**	**33.35**	**100**	**33.7**	**33.9**

**Figure 2:**
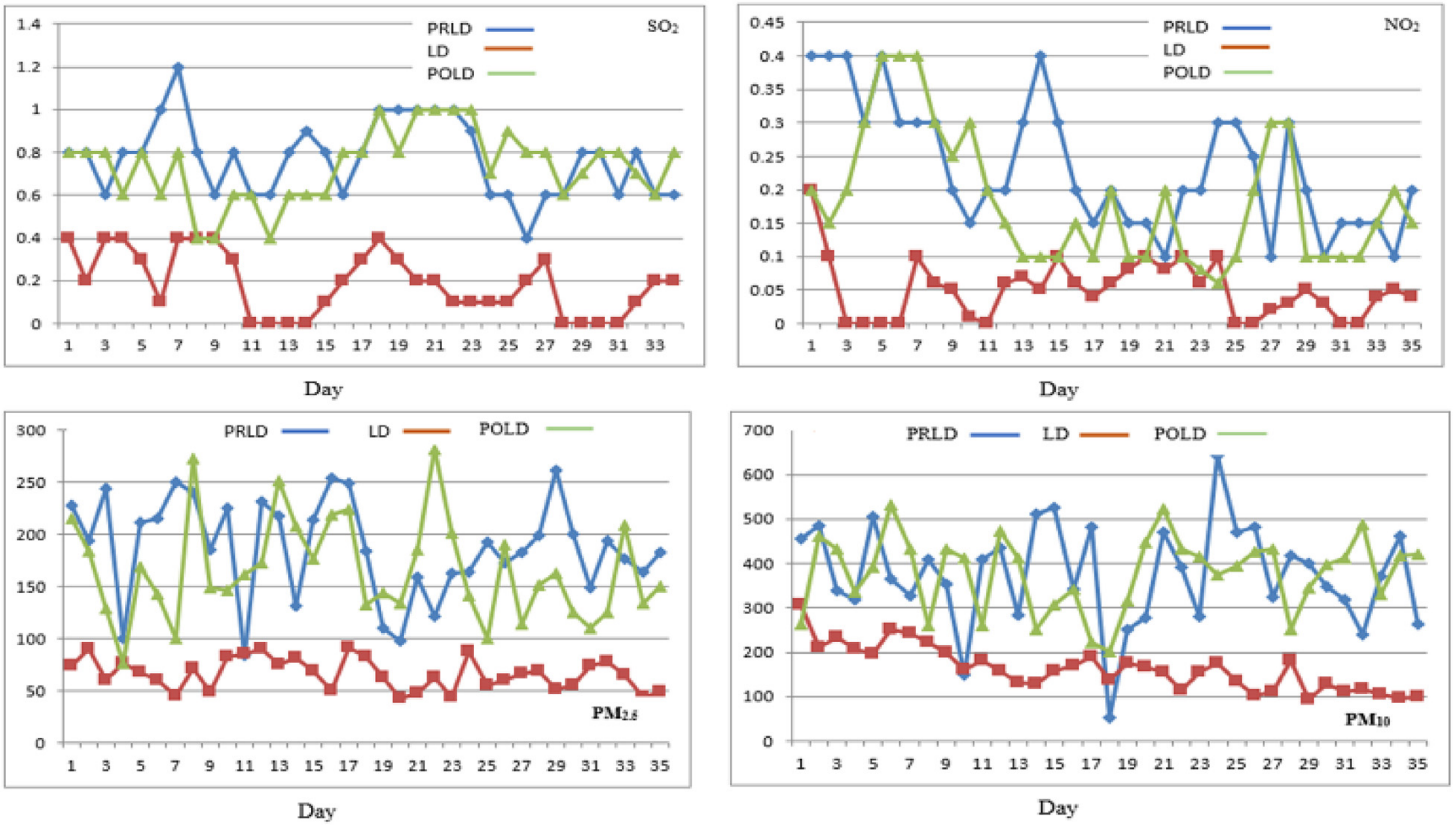
Graph showing variation of air pollutant during pre-lockdown (PRLD), lockdown (LD) and post-lockdown (POLD) periods.

Meteorological conditions can also play important roles in the diffusion and transport of the pollutants. Prior to lockdown, windspeed, relative humidity and average temperature ranged from 4–25 m/s, 33–60% and 27–33 °C, respectively, while during the lockdown the weather conditions ranged from 1–16 m/s, 69–85% and 25–30 °C, respectively and after the lockdown, the conditions ranged from 3–21 m/s, 30–54% and 26–35 °C, respectively. Before and after the lockdown, there were not only higher wind speeds but the air was drier based on the low percentages of relative humidity during these periods. This favoured the diffusion of particulate matter and other pollutants, hence high concentrations of the pollutants before and after the lockdown periods. Little changes in weather condition could amount to variation in the concentrations of the pollutants in large proportion (Sharma et al., 2020). Temperature changes, wind speed and direction, air humidity etc. can greatly influence the changes in the concentration of air pollution (Filonclyk and Peterson, 2020). Unfavourable weather conditions can enhance the accumulation of harmful pollutants on the surface layer of the atmosphere (Wang et al., 2020). It is important to note that the same trend was observed from the two different data as there was a reduction in the concentrations of the pollutants during lockdown period. Furthermore, despite reduced concentrations of the contaminants as captured from the satellite, the levels of the pollutants were still above the recommended permissible limits. This is further explained by time series of the measured pollutants (Figure 2). It was observed that all the time series have seasonal variation. Apart from the restrictions placed on movements and industrial operations, concentrations of the pollutants could also be linked with seasonal variation which in turn, is connected to the activities and the climatic conditions of the area (Yoo et al., 2014; Fuwape et al., 2022).

Pre-lockdown sampling period was from December 2019 to February 2020 (dry season), while sampling period during lock down took place from May through July, 2020 (raining season) and the post lock down sampling ran from October to December, 2020 (dry season). Generally, during dry season, (pre-lockdown and post lockdown periods in this study), climatic conditions are suitable and friendly for combustion processes, biomass burning, diffusion of particulate matter (or movement of dust) as well as other human activities that contribute to pollution load in the air (Held et al., 2012), hence high concentrations of these pollutants during these periods. On the other hand, in raining season, precipitation washout allows for the mixing of the air pollutants with precipitation; and this brings about lowered concentration of the pollutants in the atmosphere.

The models, developed to show relationship between time series of the pollutants and the metrological variables using step-wise regression are presented in Table 4.

**Table 4:** Validated models for the pollutants and climatic variables.

S/N	Models	R^2^ (adj)
1	**Pre-lockdown**	
	SO_2_ = 5.15 − 0.70 Wn + 0.04 Wn^2^ − 0.00 Wn^3^	0.83
	PM_2.5_ = 22.7 − 2.61 Wn + 0.18 Wn^2^ − 0.01 Wn^3^	0.79
	PM_10_ = 2061 − 72.6 Rh + 0.85 Rh^2^ − 0.01 Rh^3^	0.81
**2**	**Lockdown**	
	SO_2_ = 25.5 − 0.69 Rh + 0.01 Rh^2^ − 0.00 Rh^3^	0.76
	PM_2.5_ = −85.6 + 15.5 Rh − 0.37 Rh^2^ + 0.00 Rh^3^	0.75
	PM_10_ = 206.3 + 5.84 Rh − 0.25 Rh^2^ + 0.01 Rh^3^	0.65
**3**	**Post-Lockdown**	
	SO_2_ = 3.8 − 0.62 Wn + 0.07 Wn^2^ − 0.00 Wn^3^	0.59
	NO_2_ = −19.51 − 0.19 Wn + 0.864 Tav	0.78
	PM_2.5_ = −47.3 − 1.59 Wn + 3.32 Tav	0.65
	PM_10_ = 153.0 − 2.55 Wn − 1.06 Rh	0.52

The selected models for pre-lockdown show that climatic conditions accounted for the detection of pollutants ranging from 79 to 83% with wind speed being the major factor for SO_2_ and PM_2.5_ emissions; and relative humidity being responsible for the emission of PM_10_. Similarly, for lockdown, it was observed that the same climatic conditions (relative humidity) was responsible for 65–76% pollutants in the ambient air. During these periods, NO_2_ could not generate a model with appreciable coefficient of determination. For post-lockdown however, windspeed favoured diffusion of 78% of atmospheric NO_2_, while combinations of other climatic conditions favoured diffusion of other pollutants in the range of 52–65%. This observation is in support of earlier submission that climatic conditions affect the amount of the atmospheric pollutants detectable in the air (Abolude et al., 2022).

### 3.3 Air quality index (AQI)

Using AQI chart proposed by US EPA (2018) to determine the health risk associated with the exposure to outdoor air concentrations recorded in this study. The chart, which is based on colour, index values and level of health rating, gives rating scale for outdoor air, with low scale indicating a satisfactory and friendly air quality having little or no risk. Based on the estimation of sub-index values for each concentration vis-à-vis Table 3, it was observed that prior to the lockdown, the air quality was generally hazardous-which is an indication of emergency health warning. However, during restriction period, the air quality became unhealthy which shows a little improvement. After the lockdown period, the index value indicated very unhealthy situation. This was due to the gradual increase in the level of contamination of atmospheric air as a result of resumption of human activities. The index-based approach is crucial to alert the general public of the nature of the air in the environment and how healthy or otherwise exposure to such air could be.

## 4 Merits and limitations of the study

The study complements the existing studies by presenting a useful information on the understanding of the roles of human activities on the deterioration of air quality. This understanding is crucial towards the enhancement of environmental sustainability. In this study, there were some realistic constraints which led to a number of identified limitations. Ideally, the measurement should be taken every 1-h but this could not be done due to movement restriction experienced during sampling period, hence 24- hour measurement was considered (although appropriate interpolation was done following standard method). Also, consideration was not given to specific source contribution and direct impact of the emissions on health, further research in these areas is therefore suggested. Furthermore, only four pollutants (SO_2_, NO_2_, PM_2.5_, and PM_10_) were monitored in this study, more pollutants can be considered in subsequent studies for more details. Also, more analysis could have been done if the required software were available.

## 5 Conclusion

The impacts of COVID-19 lockdown on ambient air quality of African countries using Lagos city as a case study has been investigated in this study. The levels of pollutants before, during and after the lockdown were compared to determine the level of change due to movement restrictions. The results revealed a reduction of 74.0, 79.7, 55.0 and 58.5% in the levels of SO_2_, NO_2_, PM_2.5_, and PM_10_, respectively during the lockdown period. Although the reductions were short-lived because the deterioration of the air quality commenced after the restrictions were lifted. These reductions could be linked to the reduction in industrial and vehicular activities during the period of the pandemic. The results further revealed that though, the lockdown period witnessed huge reductions in the atmospheric emission within Lagos, air quality was still poor, as the levels of most of the pollutants were above the permissible limits and this suggests that apart from restricted activities, there are other sources of air pollution within the city which increase the pollution load in the ambient air. The overall results of this study are in conformity with other studies from other continents. The effects of poor air quality on human and environmental health calls for serious concern, especially with the growing rate of incidence of cancer-related diseases in the continent of Africa, and thus, the need for decisive actions to mitigate air pollution. To be in tandem with global best practices, cleaner technology should be advocated to ensure reduction in the consumption of fossil fuel instead of the common practice of end-of-pipe technology. It is concluded therefore that, despite the economic hardship brought about by the lock-down, it greatly enhanced the quality of ambient air.

## Declarations

### Author contribution statement

Ebenezer Leke Odekanle: Conceived and designed the experiments; Performed the experiments; Analyzed and interpreted the data; Wrote the paper.

Fakinle, B.S; Odejobi, O.J. Sonibare, J.A., Akeredolu, F.A: Conceived and designed the experiments; Contributed reagents, materials, analysis tools or data.

Akangbe, O.E. and Oladoja, O.M: Analyzed and interpreted the data; Contributed reagents, materials, analysis tools or data.

### Funding statement

This research did not receive any specific grant from funding agencies in the public, commercial, or not-for-profit sectors.

### Data availability statement

Data included in article/supp. material/referenced in article.

### Competing interest statement

The authors declare no conflict of interest.

### Additional information

No additional information is available for this paper.
